# News coverage, digital activism, and geographical saliency: A case study of refugee camps and volunteered geographical information

**DOI:** 10.1371/journal.pone.0206825

**Published:** 2018-11-08

**Authors:** Ron Mahabir, Arie Croitoru, Andrew Crooks, Peggy Agouris, Anthony Stefanidis

**Affiliations:** 1 Department of Geography and Geoinformation Science, George Mason University, Fairfax, Virginia, United States of America; 2 Center for Geoinformatics and Geospatial Intelligence, George Mason University, Fairfax, Virginia, United States of America; 3 Department of Computational and Data Sciences, George Mason University, Fairfax, Virginia, United States of America; 4 Center for Earth Observing and Space Research, College of Science, George Mason University, Fairfax, Virginia, United States of America; 5 Criminal Investigations and Network Analysis Center, George Mason University, Fairfax, Virginia, United States of America; University of Warwick, UNITED KINGDOM

## Abstract

The last several decades have witnessed a shift in the way in which news is delivered and consumed by users. With the growth and advancements in mobile technologies, the Internet, and Web 2.0 technologies users are not only consumers of news, but also producers of online content. This has resulted in a novel and highly participatory cyber-physical news awareness ecosystem that fosters digital activism, in which volunteers contribute content to online communities. While studies have examined the various components of this news awareness ecosystem, little is still known about how news media coverage (and in particular digital media) impacts digital activism. In order to address this challenge and develop a greater understanding of it, this paper focuses on a specific form of digital activism, that of the production of digital geographical content through crowdsourcing efforts. Using refugee camps from around the world as a case study, we examine the relationship between news coverage (via Google news), search trends (via Google trends) and user edit contribution patterns in OpenStreetMap, a prominent geospatial data crowdsourcing platform. In addition, we compare and contrast these patterns with user edit patterns in Wikipedia, a well-known non-geospatial crowdsourcing platform. Using Google news and Google trends to derive a measure of thematic public awareness, our findings indicate that digital activism bursts tend to take place during periods of sustained build-up of public awareness deficit or surplus. These findings are in line with two prominent mass communication theories: agenda setting and corrective action, and suggest the emergence of a novel stimulus-awareness-activism framework in today’s participatory digital age. Moreover, these findings further complement existing research examining the motivational factors that drive users to contribute to online collaborative communities. This paper brings us one step closer to understanding the underlying mechanisms that drive digital activism in particular in the geospatial domain.

## Introduction

Recent studies (e.g. [1]) indicate that the general public is increasingly using a combination of online platforms (e.g. news websites/apps and social media) and traditional news avenues (e.g. local and cable TV, radio, and print newspapers) to get its news. This results in a novel news ecosystem, supporting highly and actively engaged news consumers [2–4]. Within this news ecosystem, cyberspace is complementing traditional sources to communicate news [5–7], raise public awareness [8,9], and form and shape opinions and agendas [10–12] across a diverse range of issues.

This highly participatory cyber-physical news awareness ecosystem is fostering digital activism. Its manifestations range from encouraging and enabling the crowd to generate, publish, and disseminate focused information, to fostering the formation of online communities that jointly pursue activities in the cyberspace, and often the physical space too [13]. We are repeatedly witnessing this in the use of social media to support political activism in the streets of cities across the globe [14–19], the establishment of peer-to-peer information exchange communities [20,21], or even the formation of online communities to support cyberspace projects like Wikipedia [22]. While this powerful cycle linking information consumption with the raising of awareness and the resulting digital activism will continue to be essential to the future of our society, the mechanisms that drive this process still remain fuzzy.

One popular expression of such digital activism is in the form of volunteered contributions to online collaborative activities. In order to better understand the motivating factors that drive such activities, certain studies have focused on individual platforms separately. Nov [23] built on earlier work of Clary [24] that had identified the major motivational functions that drive volunteerism overall (e.g. altruistic values, social interaction, enhancing one’s understanding and career-related benefits) to show that these general motivations apply to Wikipedia contributions as well. Xu and Li [22] classified these functions under two broad categories, namely the will to contribute content, and the interest in participating in that online community. Budhathoki and Haythornthwaite [25] examined the motivational factors that drive contributions on OpenStreetMap (hereafter referred to as OSM in this paper), a freely accessible and editable map of the world [26–28]. That study reported important positive factors relating to personal, yet the shared need to contribute to open source projects, the co-integration of individuals into open-source and geographic knowledge communities, along with the need to be attentive to participation taking places within the OSM community. It is also important to note that the motivation for contributors within these different communities can vary [29,30].

The above studies have been crucial in providing a better understanding of the various factors that drive people to become digitally active in online collaborative communities. However, relatively few studies have examined digital activism in the context of the previously described broader news awareness ecosystem. Zastro [31], for example, noted that during the Haitian earthquake in 2010, volunteer mappers used a combination of different news media (i.e. news reports, social media and text messages from survivors) to collect information on the damaged status of buildings. This information was then added to the OSM platform. Westrope et al. [32] examined the possible impact of media coverage on mapping activities following Typhoon Haiyan in the Philippines in 2013. That study showed that in areas that received substantial media coverage (e.g. Tacloban City) 92% of the surveyed buildings had over-represented damage reported in the OSM edits. In contrast, in areas that received less media coverage only 56% to 76% of the buildings had over represented damage reported in the OSM edits. As the authors of that study suggest, the large over-representation in damage buildings in Tacloban City could be attributed, at least in part, to the priority given to this city by the Humanitarian OpenStreetMap Team (HOT) OSM community. In a related study, Dittus et al., [33], compared OSM activity for various Humanitarian HOT events, showing that the two most publicized media events, Typhoon Haiyan, and the Nepal earthquake in 2015, had the largest newcomer mapping recruitment rates. The authors of that study suggested that news coverage of these events may have assisted in attracting a much larger number of newcomers. Similar findings of the influence of media coverage and an increase in newcomer enrollment in the OSM platform were reported by Begin et al., [34]. While these studies shed some light on the possible relation between OSM activity and news media coverage, a more in-depth analysis of this relation is still needed.

The pursuit of a deeper understanding of the relationship between the different components of the complex news awareness ecosystem and digital activism is becoming a substantial research challenge. Wikipedia edit patterns, for example, have been compared to breaking news [35], and news media coverage of an issue to increases in searches for and edits of related articles in Wikipedia [36]. Such patterns have also been used to define entity-specific news tickers and timelines [37], with page views further used to detect popular topics related to users’ interest [38]. Google Trends [39], a tool for measuring public agenda [40,41], has also been found to be useful for quantifying trading behavior in financial markets [42,43], for disease surveillance and health care research [44,45], and as a tool for behavior analytics, such as predicting non-cigarette tobacco use [46]. Moreover, Ratkiewicz et al., [47] show that bursts of online search volume activity extracted from Google Trends tend to be correlated to similar bursts of editing activity on Wikipedia. Such studies highlight just how complex this news awareness ecosystem is.

In the context of this paper we use the term news awareness ecosystem to refer to the ensemble of sources, ranging from traditional newsrooms to grassroots citizen journalism, that provides the public with possible exposure to news, and from which public awareness can emerge. A key characteristic of this ecosystem is that rather than being monolithic it is multifaceted, multimodal, and evolving. This term builds on the idea of the “news ecosystem” that has become prevalent in recent years. While an agreed upon definition of this term is still lacking, several attempts towards the construction of such a definition have been made. For instance, Anderson [48] provides a brief genealogy of the term and its origins, and discusses several concepts that may support the construction of such a definition. Alternatively, Picard [49] provides a detailed discussion of the forces that reshaped news journalism into what is referred to as the new (news) ecosystem.

Taking a more non-contemporaneous view of the news cycle, Nghiem et al., [50] showed that, depending on the specific topic, news coverage can be responsive to or lag an occurring trend (as defined by Google Trends search volume data). In some cases, albeit very few, a build-up of news activity was also followed by a decrease in search volume. Such responsive behavior of news patterns has been documented for trending topics in social media as well [51,52]. Althoff et al., [53] compared trending data from Google (Search, News and Trends), Twitter and Wikipedia to show that combined, these different data platforms can be used to forecast trending topics, even though temporal activity patterns (comprising spikes and cumulative build-up) tend to vary across these platforms. Other work by Al Emadi et al., [54] showed the strong response of the online mapping communities in the aftermath of natural disasters, e.g. in the form of increased online volunteering activity in the MicroMapper [55] crowdsourcing platform.

Digital activism and geography have long been intertwined, giving rise to a range of online user activities, which in the context of this paper we refer to as geo-activism (in the remainder of the paper we will use the term digital- and geo-activism interchangeably). From crowdsourcing crisis information mapping, to support disaster relief and recovery efforts [56,57] and collectively editing geographical data in OSM [27], volunteered geographical information (VGI) has emerged as a substantial new mechanism for the general public to contribute geographical content by mapping roads, buildings, and other artifacts [58–60]. At the same time, it is important to recognize that the general public is also increasingly engaged in geo-activism while being exposed to the news media. As suggested in previous studies [61–63], many forms of online news media have the power to shape public opinion, affect other media sources, and foster engagement.

Consequently, a key premise of our work is that geo-activism should be considered in the context of the news media rather than as a separate, independent phenomenon, in order to advance our understanding of the mechanisms that drive it. This would allow us, for example, to explore how the ebb and flow of information in the public media sphere affects digital geo-activism. At a time where VGI is establishing itself as a rich supplementary—and sometimes only—source of geospatial information [64, 65], a better understanding of the mechanisms driving geo-activism will allow us to further harness its power.

Accordingly, our objective in this paper is to study links between media coverage and geo-activism by focusing on the question of *geographical saliency* within the news awareness cycle. The basic argument is that news features of broad community interest but with a certain, narrow geographical footprint build up over time awareness and interest for that location, leading to bursts of volunteered geographical contributions for it. We pursue this task by comparing patterns of online volunteered geographical edits in OSM to relevant news stories, and to corresponding edit patterns in Wikipedia. Therefore, the research question at the core of this paper is whether extreme instances of public awareness deficit or surplus (resulting from sustained relevant trends) tend to trigger digital geo-activism bursts.

Towards this goal, we present in this paper a case study that focuses on several refugee camps around the world to determine whether salient features in news media affect digital activism in OSM. This line of inquiry allows us to examine the possible association and interconnectedness between macro level global awareness and news coverage and digital activism, with a hyperlocal geographical focus.

A number of factors render refugee camps particularly suitable for such a study. First, they match very well the above-mentioned motivational factors that drive digital activism as they were identified by Nov [23]. Second, compared to previously studied events like natural disasters, refugee camps differ in the sense that they are not abrupt events that occur over a period of few hours (e.g. earthquake) or days (e.g. flooding), which would render the relationship between media coverage and digital activism trivial, as event, news coverage, and response practically coincide. Instead, these refugee camps are set up and operate over a period of years [60] and public awareness to them builds over time. Third, they offer the advantage of having a geographically distinct footprint in terms of size and location relevant to their surroundings while being associated with a narrow thematic focus at the same time (unlike large cities, which may have numerous themes occurring at the same time). Finally, in the aftermath of massive displacement of populations due to civil war or conflict (e.g. the Syrian crisis that began in 2011 and other on-going ethnic tensions), refugee camps represent a topic that is becoming increasingly important in terms of media coverage and public awareness [66].

The remainder of this paper is organized as follows. In Section 2, the datasets and methods used to address our research question are presented followed by an in-depth discussion of the results in Section 3. Finally, Section 4 provides a discussion of our results and concludes with an outlook of future work.

## Materials and methods

### Refugee camps

**Fig 1:**
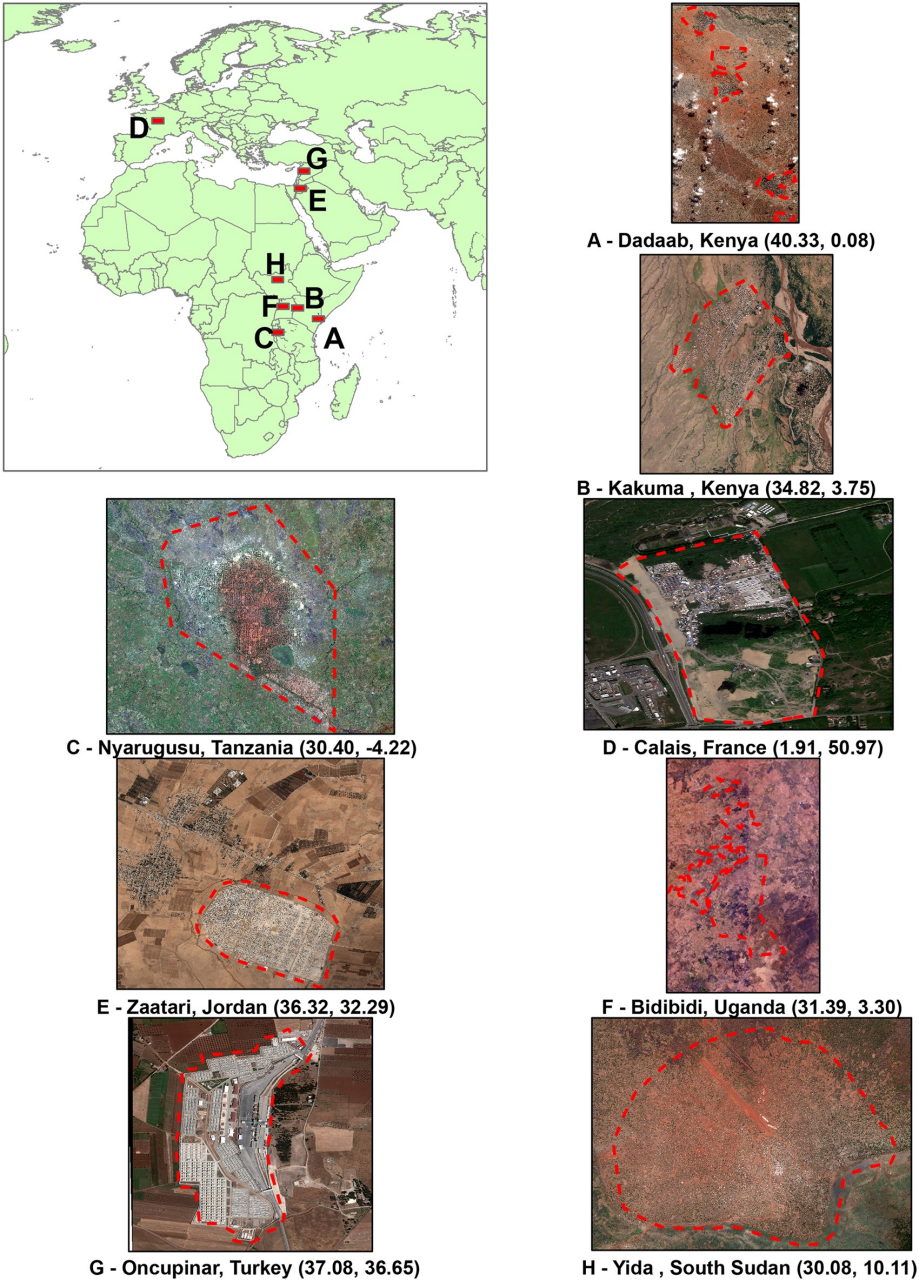
Study areas (centroid location of camp). Satellite image courtesy of the DigitalGlobe Foundation.

The current concentration of refugees is highest in African and Middle Eastern countries compared to other world regions [67]. These regions also contain the most populous camps as well [68]. Accordingly, a set of 8 refugee camps was selected for our study, located in Africa, Middle East, and Europe, as shown in Fig 1. In addition to reflecting the global distribution of refugees, these camps offer diversity in terms of their size, population, and date of establishment. Camp age (date of establishment) is of particular interest, as it allows us to study camps at different stages of news cycles and interest.

Table 1 shows more detailed information for each camp including their established date and camp status, their size (area), population, and population density (See S1 File for references). As expected, larger camps tend to host more refugees, and the correlation between camp size and population has a Pearson *r* value of 0.70 with a p-value of 0.03 (with Kakuma being the one exception). However, in general, there is no clear observable association between the age of the camps and their population density or size.

**Table 1:** Refugee camps.

Camp	Country	Established Date/Status	Area (km^2^)	Population	Population Density (People per km^2^)
Dadaab	Kenya	1992 with an addition in 2011 / Active	40.7	242,998	5,970
Kakuma	Kenya	1991/Active	6.4	171,085	26,732
Nyarugusu	Tanzania	1996/Active	25.9	78,519	3,032
Calais	France	2015/Closed on October 2016	0.61	9,000	14,754
Yida	South Sudan	2011/Active	20.4	55,012	2,697
Bidibidi	Uganda	2016/Active	360.9	270,000	748
Oncupinar	Turkey	2012/Active	0.71	15,000	21,127
Zaatari	Jordan	2012/Active	6.1	79,827	13,086

### Data sources

To analyze patterns of geo-activism as it relates to these refugee camps, we used the patterns of relevant information contributions in OSM. OSM is a prototypical example of a VGI platform allowing anyone to map features on the Earth’s surface. It was launched in July of 2004 and presently, as of August 2018, has almost 5 million registered users [69]. While being general in its scope, OSM has grown over time to be a particularly rich source of geographical data, and especially so in support of humanitarian response activities. Some recent examples include OSM contributions relevant to the Ebola outbreak in West Africa in 2014 [70], the Nepal earthquake in 2015 [71], and slums in Sub-Saharan Africa [65].

As OSM is a collaborative platform of information contribution, it tends to exhibit activity patterns similar to those found in other non-geographical platforms. For example, a common thread in many online collaborative communities is that the level of participation is not even: few users tend to contribute massive amounts of information, whereas the large majority tends to contribute less. Previous studies have confirmed that this pattern is also present in the OSM community [72,73]. Similar patterns of contributor activity have also been found with other online crowdsourcing communities such as Wikipedia [74–77], which are often characterized by bursts of activity [78,79]. When it comes to OSM, the spatial and temporal frequency of contributions can vary based on numerous factors, including, the diversity of contributors [27,80], issues associated with the digital divide [81–83], the social structure of contributor communities [84–85], the direct intervention of social groups such as mapping parties [86,87], and bulk imports into the OSM platform [88], among others.

For the purpose of our study, we used OSM data that were extracted from the *planet history file* (https://planet.openstreetmap.org/planet/full-history), which contains all OSM edits for the entire world. An OSM edit herein is defined as any create, delete or modify operation to any OSM node, way or relation feature. The OSM planet history file also contains other useful information, such as the location (latitude and longitude) of each node, object versions (from October 2007 onwards), contributor user ID, and timestamp. Refugee camp OSM data were clipped from the history file using spatial and temporal parameters. In order to delineate the footprint of each camp we use polygons that demarcated their spatial extent, and use these polygons to select the corresponding edits. Camp polygons were manually traced using high resolution satellite imagery over each camp. In terms of time window, we selected edits during the current decade (01/01/2010 to 05/31/2017). From among all our camps, only Bidibidi had edits (only 2) prior to our study window, and so in practice our study addresses the whole OSM history of these camps.

In conjunction with the collection of OSM edit activity, edit activity was collected for the Wikipedia page entries that correspond to each of the refugee camps in Table 1. A Wikipedia edit in the context of this research refers to any change (i.e. creation, modification or deletion operation) made to the content of a Wikipedia page. In our study the edit activity of each page was collected from the history page of each camp’s Wikipedia page using the same time window parameters noted above for OSM. Only English pages were used in this study, a caveat that will we will later revisit in the Discussion section. Wikipedia is the world’s largest free online encyclopedia, allowing anyone to create new and edit existing articles. As of August 2018, more than 34 million users were registered with almost 46 million pages, and with the number of entries now approaching 6 million [89]. A key motivation to explore the edit activity of Wikipedia alongside the edit activity of OSM is that while both platforms rely on digital activism through crowdsourcing efforts, the former focuses on digital activism in a more general sense while the latter focuses specifically on geo-activism. This difference will enable our analysis to compare and contrast digital activism patterns in two substantially different crowdsourcing environments.

In order to analyze relevant news media activity to OSM edit activities we also collected relevant data from Google News and Google Trends. While Google News conveys expressions of media coverage, Google Trends is considered here as a proxy for public interest. News coverage data were captured from Google News [90], an online news aggregator for up-to-date news stories from all over the world. As of 2012, Google stated that it draws from more than 50,000 news sources with more than one billion unique users connected each week to its news content [91]. Prior research has also compared news extracted from Google with other platforms such as LexisNexis, showing that Google News provided broader worldwide coverage than its counterparts [92]. Google News articles were searched using the keywords “*name of camp*” AND “*refugee*” to help filter non-relevant stories. Articles were further filtered manually by the authors as needed, to remove irrelevant data. Between 6% and 26% (average of 13%) of articles were removed during the data cleaning process. Filtering was initially done by one of the authors who collected the data, and later on two other authors assisted to further remove articles that were deemed non-relevant. The remaining relevant news articles were then binned into daily counts.

**Fig 2:**
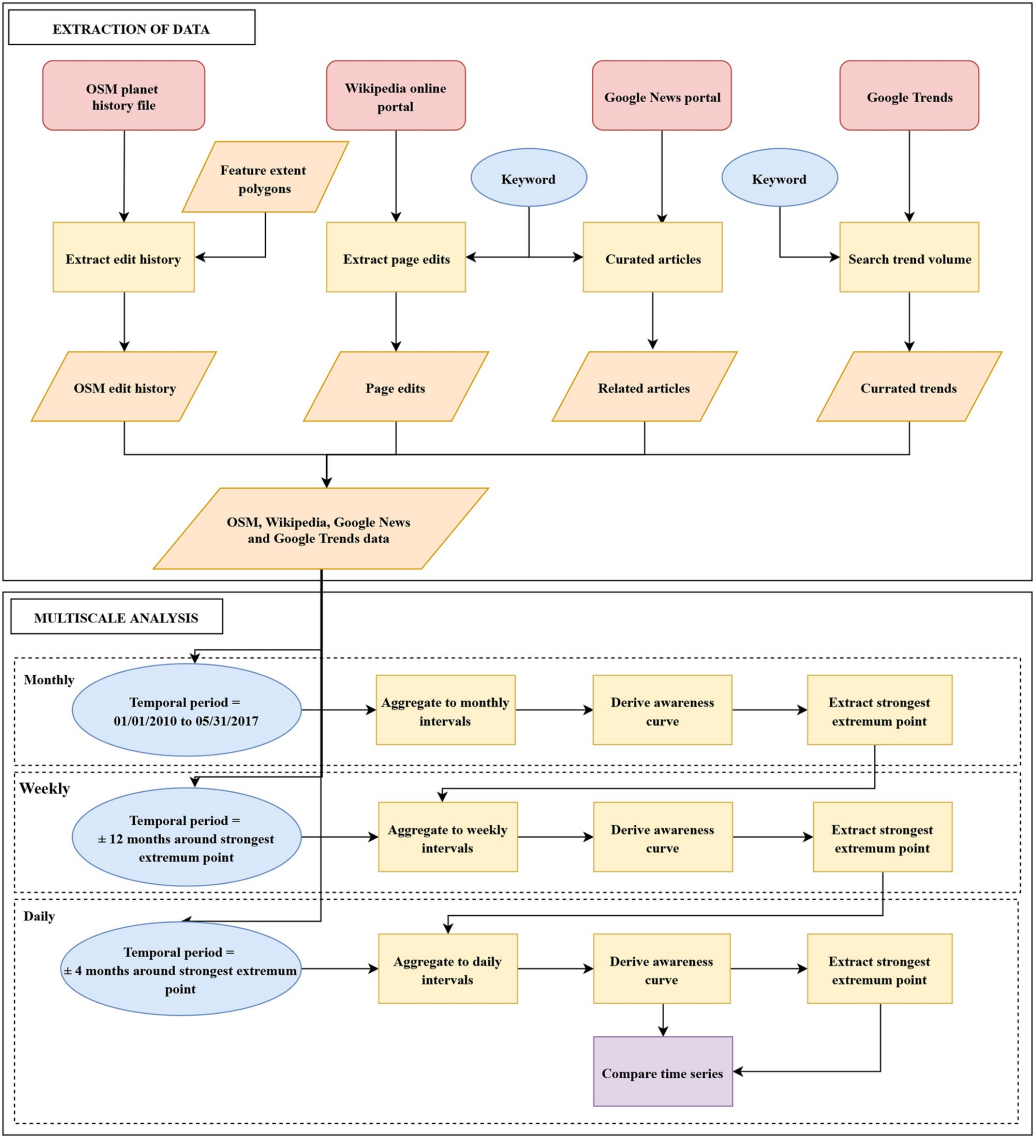
Data extraction and preprocessing. In the data extraction step, data on OSM, Wikipedia, Google News, and Google Trends is collected from their various online platforms. The multiscale analysis step involves a progressive refinement approach: Data is first examined at the monthly level and the strongest extremum points on the awareness curve are identified. These points are then assigned a score that is comprised of its magnitude and a weight that is proportional to the duration of consistent trend along the awareness curve prior to the detected extremum point. A search time window is then defined on the awareness peak with the highest score and used for working with data at the weekly analysis level. A similar approach is used to identify a search window for working with data at the next (finer) temporal granular analysis level.

To capture information on public interest, we used Google Trends data. Google Trends provides information on how often a particular keyword was searched using Google’s web search engine. This data has also been used in previous studies as a proxy measure for public interest in a variety of topics [40,50,93,94]. In order to access Google Trends data for each camp, we used the corresponding online portal and the camp’s name as the keyword, to capture searched volumes for each camp. This data provided by Google, is available as a normalized series of values between 0 and 100, based on the popularity of users’ search interest within the specified temporal range. A summary of the various steps used to extract and preprocess the different data sources (OSM, Wikipedia, Google News, and Google Trends) are presented in Fig 2. Further, in S2 File, we show the total number of OSM edits, Wikipedia edits and Google News articles at the daily level for the period 01/01/2010 to 05/31/2017. With respect to Google Trends, data at the daily level is limited to a temporal search window within 269 days. Following this, the data is provided at weekly and then the monthly aggregated level depending on the temporal search window. As such, the Google Trends data is not included in S2 File. An initial visual comparison of the data at the daily level did not reveal any apparent patterns across the different data sources.

### Overview of multiscale and multiplatform comparison approach

Given the objective of this research, our approach is based on using four basic measures to explore the possible associations between news media coverage and crowdsourced activity: Google Trends index, Google News volume, OSM edits activity, and Wikipedia edits activity. While the first two measures serve as a proxy of the general public’s interest and availability of news around specific themes, the latter two measures serve as a proxy of the level of digital activism around specific thematic features over space and time. A key premise of our approach is that comparing and contrasting these measures can provide additional insight into the possible relationship between the ebb and flow of news and activity patterns in digital activism in platforms such as OSM and Wikipedia.

While gauging the levels of digital activism is relatively straightforward (e.g. tracking the number of edits in Wikipedia), measuring the ebb and flow of news in the media ecosystem is more challenging as there is no such single readily available measure. To overcome this issue, we propose to consider the difference between the normalized Google Trends index and the normalized Google News metric as a measure of the overall *public awareness* with respect to a specific theme (or a set of themes). Here, we refer to the term awareness simply as the “knowledge that something exists, or understanding of a situation or subject at the present time based on information or experience” [95]. The reasoning behind using this difference measure is that while Google News represents the availability of news (i.e. information) regarding a theme, Google trends represents the public’s active pursuit of information about the theme. As news stories emerge, evolve, re-emerge, and eventually subside in the media, the public’s information seeking activities may increase, decrease, or remain unchanged over time [50]. Consequently, a surplus (or deficit) of *public awareness* can be built-up over time through the availability (or unavailability) of news stories that consistently increase (or reduce) the public’s online information seeking activities. A prolonged period of awareness surplus growth would be one where the normalized metrics of Google Trends grow faster than the corresponding metrics of Google News, implying that public interest on the topic grows faster than news coverage. Conversely, a prolonged period of awareness deficit would be one where news coverage outpaces public search interest. In this context of this paper, a min-max approach was used to rescale all four data metrics to values between 0 and 1.

The ability to track trends in public awareness overall enables us to explore the potential associations between such trends—and in particular trend changes—in awareness and digital activism. Specifically, our interest lies in the question of *how are trend changes in public awareness related to user activity in OSM and Wikipedia*? The emerging argument from this question is that media coverage drives participation, by informing the general public of evolving/developing situations, thus setting an agenda and leading to digital activism. Since such a mechanism can occur at different time scales, our approach is based on consecutively examining the different measures at 3 levels of time granularity, namely monthly, weekly and daily, in order to identify possible associations at the finest temporal granularity considered here. Starting from the monthly level, extremum points in public awareness activity are detected, and the strongest extremum point is identified. The time stamp of this point is then used together with a search time window around it to define a new search interval at the next (weekly) granularity level. This process is then repeated for identifying a search time window at the daily time granularity, in which possible relations between public awareness trend changes and OSM and Wikipedia activity are explored. A more detailed description of the extremum points detection and selection process is provided below.

Our analysis process begins by examining the public awareness curve at the monthly level and then proceeds using a progressive refinement mechanism towards an examination of data at the daily level. In order to capture broad trends in public awareness level, we model the public awareness data using multivariate adaptive regression splines (MARS). MARS is particularly suitable for this type of data since it makes no assumptions about the underlying distribution of the data and provides an intuitive approach for understanding the intrinsic complicated data mapping in high-dimensional data patterns [96]. MARS is represented as a combination of basis functions expressed as [97]:

$$y=\beta_{0}+\sum_{m=1}^{M}\beta_{m}h_{m}(X)$$ (1)

where *y* is the dependent variable (i.e. our public awareness measure), *X* is the independent variable (time), *β*_*0*_ is the intercept parameter, and *β*_*m*_ is the coefficient applied to each basis function *h*_*m*_*(X)*, which are summed over *M* non-constant terms used for defining the number of basis functions. In this case, *M* is determined in a data-driven manner based on the optimum number of basis functions required to fit the data. While MARS supports polynomial basis functions, in our implementation linear basis functions are used for simplicity.

Using the derived MARS model, extremum points in the public awareness curve are detected by estimating the second derivative *f*″(*x*) as a central finite difference approximation:

$$f^{\prime \prime }(x)\approx \frac{f(x_{i}+\Delta x)-2f(x_{i})+f(x_{i}-\Delta x)}{{\Delta x}^{2}}$$ (2)

where *x*_*i*_ represents the *i*^*th*^ value of *x* in the data series defined by the predicted values in Eq 1, *Δx* is the step size in temporal units (i.e. monthly, weekly or daily dependent on the scale of analysis) around point *x*_*i*_, where *i* = 0,1,2,…,*n*. At each temporal granularity level *Δx* is set to 1 time unit, e.g. at the monthly level a *Δx* value of 1 month is used.

As the detection of extremum points along the public awareness curve may result in multiple local maxima or minima points, a pruning process is applied in order to capture the most prominent extremum points. This process begins by identifying all extremum points and then for each point suppressing other weaker extremum points (in terms of their absolute magnitude) within a local window centered around each top extremum point. In our analysis this was set to ±1.5 months, ±1 week, and 0 for the monthly, weekly and daily granularity, respectively. In the case of Google Trends, this data was first captured at weekly granularity to identify local windows around each extremum point at the monthly analysis level. Following this, at the next level of analysis (i.e. weekly), the Google Trends data was captured at daily granularity. No local windows were used at the daily analysis level.

Following the pruning process, each of the remaining extremum points (in terms of their absolute magnitude) is assigned a score that is comprised of its magnitude and a weight that is proportional to the duration of consistent trend (positive or negative) along the awareness curve prior to the detected extremum point. This weighting scheme is guided by previous work [98], which suggested that the longer a theme is circulated within the media, the greater its potential for influencing the public agenda (which may result in digital activism). Based on these calculated scores, the awareness peak with the highest score (strongest extremum point) is selected, and is then used for defining a search time interval *ΔT* at the next (finer) temporal granularity level.

The selection of *ΔT* in our analysis is guided by prior research on agenda setting theory in communication [99], in which the question of the time that it takes for the public to respond to news stories was explored. Such prior work suggested that it may take as little as a few days [100] to as much as 2 to 6 months [98,101] for changes in the media agenda to become fully realized into public agenda. In other studies, e.g. [93], a period of 50–70 days was suggested. Informed by these prior studies, our analysis is based on repeating the search for the for the highest scoring awareness peak using a progressively refined *ΔT*. Specifically, *ΔT* was set to ±12 months and ±4 months around each highest scoring awareness peak in the monthly and weekly time granularity, respectively.

As our objective relates to the comparison of the awareness curve to the two crowdsourcing platforms, it is also necessary to detect extremum points in the OSM and Wikipedia edit activity data. For convenience, and due to the overall stepwise nature of this data, we consider the cumulative edit activity time series for these two platforms and approximate each edit activity curve using the Douglas-Peucker line simplification algorithm [102]. Then, using the simplified edit activity curves, we detect the top 5 extremum points ranked by their overall magnitude. In some cases, fewer than 5 significant peaks were extracted as a result of the line simplification process.

## Results

Given the objectives of this paper and the proposed analysis approach, this section summarizes the results that were derived along three main themes. First, we outline the key trends in the edit activity for the eight camp sites. Following this, we present the results of an analysis of the edit activity patterns in the local context of each camp site. Finally, we examine how trends in public awareness relate to OSM and Wikipedia edit activity patterns.

### Edit activity trends in OSM

The overall number of OSM edits for all 8 refugee camps for the period 01/01/2010 to 05/31/2017 is shown in Table 2. Comparing these numbers with the data in Table 1 reveals a strong correlation between camp size (spatial extent) and number of OSM edits, with a Pearson *r* correlation of 0.89 and a p-value of 0.002.

**Table 2:** Total OSM edits per camp for the study period (01/01/2010–05/31/2017).

Camps	Total OSM Edits
Dadaab	31,283
Kakuma	11,147
Nyarugusu	74,416
Calais	5,965
Yida	84,053
Bidibidi	198,916
Oncupinar	708
Zaatari	69,981

**Fig 3:**
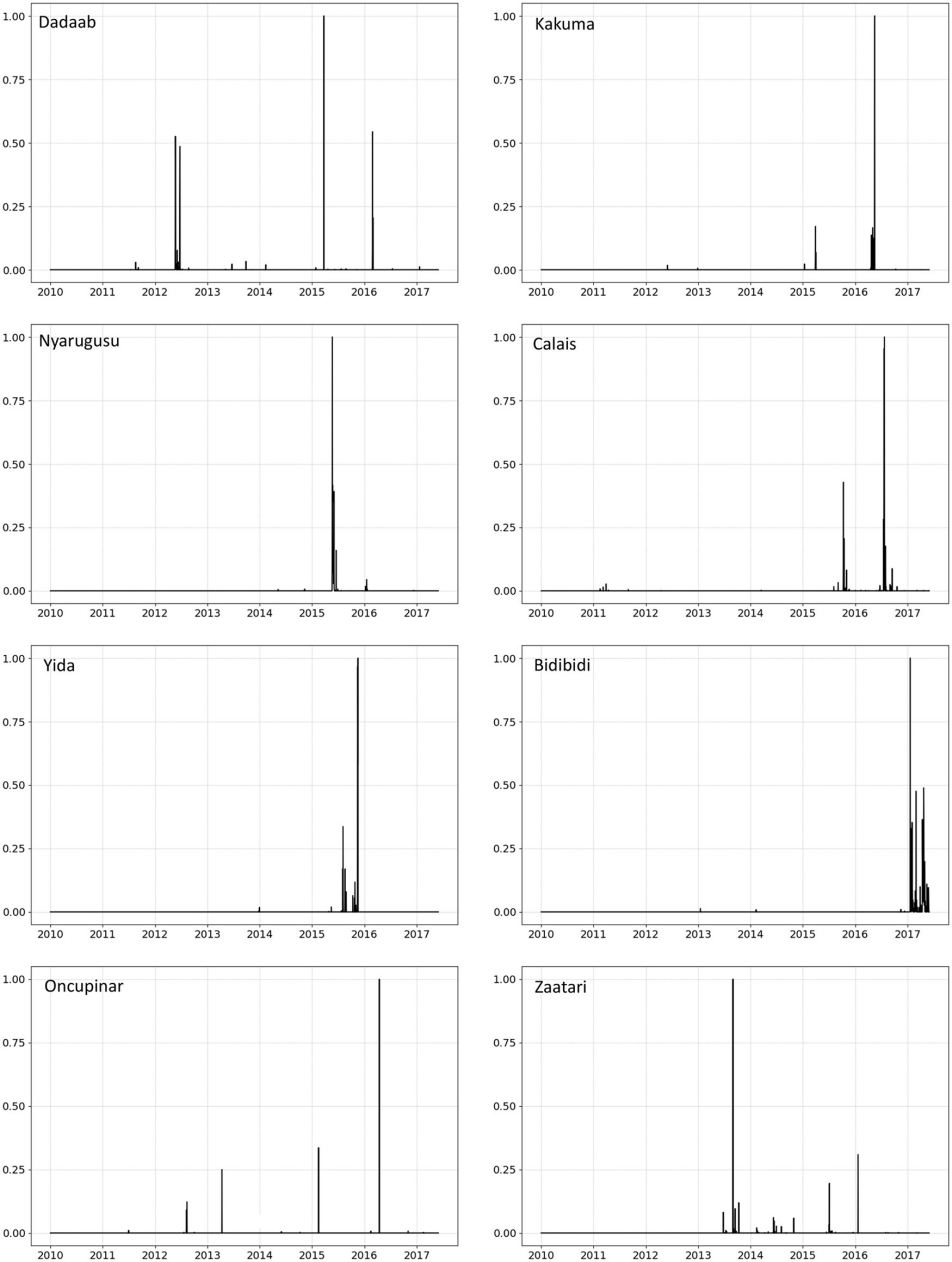
OSM edit patterns for the 8 camps studied (01/01/2010–05/31/2017).

In order to delve into the driving forces behind public participation in OSM we further look at the temporal variations of these contributions. Fig 3 shows the patterns of temporal activity in OSM edits for the 8 camps selected. The data in Fig 3 has been normalized to values between 0 and 1 to account for different scales of edit activity between camps.

Fig 3 shows that edit activity in OSM tend to occur in bursts (spikes in the charts), similar to the what was observed in Wikipedia [47]. This observation is consistent with Barabasi [103], who suggested that such an activity pattern tend to follow a non-Poisson pattern, which is common in human activities in general. Following this theory, edit activity bursts could then be seen as following a natural pattern of contributors’ interest (as a measure of priority) in mapping refugee camps. Furthermore, as such OSM edit activity bursts represent epochs in time in which users’ activity was focused on specific localized camp sites, one could argue that these bursts represent instances when these sites become salient features/attention landmarks [100] in geographic space. A more detailed discussion of this saliency property is provided in the next section.

In addition, it is interesting to observe that the OSM edit activity bursts in Fig 3 do not tend to coincide in time across camps. This is expected as it is rather unlikely that users that edit different camp sites will do so at the exact same time, and suggests that geo-activism in OSM around this theme tends to be asynchronous across different locations. This asynchronous nature could also be attributed, in part, to the specific history of each camp site. Some camps, such as Bidibidi, exhibit OSM edit activity bursts towards the end of our study period, while more established camps, such as Dadaab, exhibit several bursts of activity spread throughout the study period. For camps such as Nyarugusu, a single distinct edit activity burst is apparent. Finally, there were no clearly observed co-occurrences of OSM edit activity in Fig 3 and camp establishment dates as noted in Table 1.

### Geographical saliency: Camps as local activity hotspots

**Fig 4:**
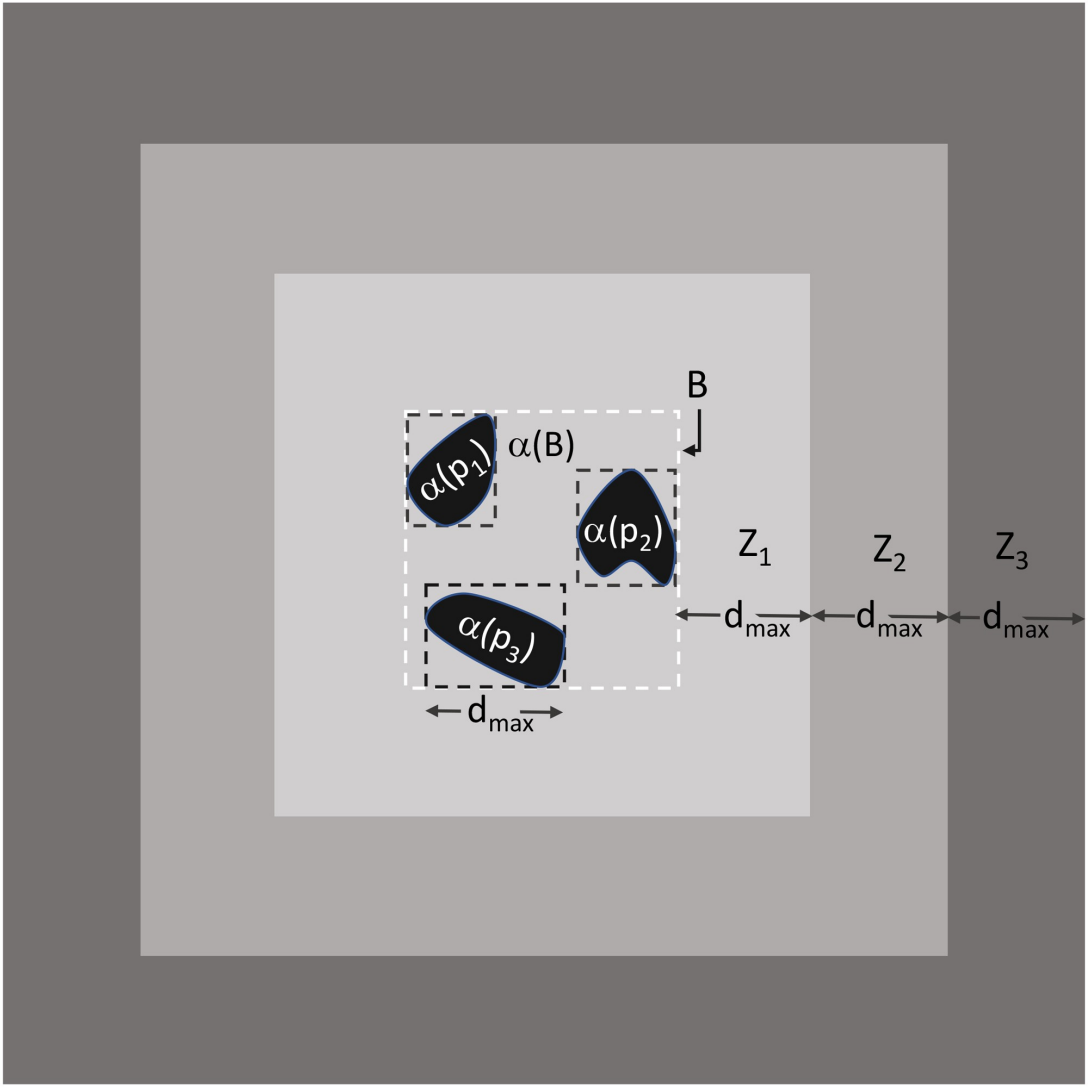
A schematic example of a camp (aggregate of black polygons), its minimum bounding box (dashed white line B) and its surrounding zones Z_1_ (light gray), Z_2_ (aggregate of light and mid gray), and Z_3_ (aggregate of Z_2_ and darkest gray).

In order to assess the degree to which a refugee camp becomes a local geographical salient artifact, overshadowing interest on its immediate surroundings, we evaluate the extent to which OSM editing activity within their boundaries exceeds the editorial activity in their immediate surroundings. In that sense, camps then become local activity “hotspots” of digital geo-activism. In order to estimate the OSM editing activity both within and around the camps, we define a set of 4 zones for each camp: a camp zone Z_0_ and 3 surrounding aerial zones, Z_1_, Z_2_, and Z_3_. The relationship between camp and surrounding zones is shown Fig 4.

Formally, zone Z_0_ is defined as the spatial footprint of the camp, as captured by the set of (one or more) polygons P = {p_1_, p_2_, …, p_n_} (n≥1) that delineate it. Based on this, zone Z_0_ is defined as:

$$Z_{0}=\cup \alpha (p_{i}),i=1,2,\ldots ,n$$ (3)

where α(p_i_) is the area enclosed by polygon p_i_, and ∪ is the union operator. Given P, the set of minimum bounding boxes of p_1_, p_2_, …, p_n_ are then derived, and the maximum side length (width or height) d_max_ among all bounding boxes is found. In addition, the minimum bounding box B that encloses P is also derived. Using d_max_ and B the zones Z_1_, Z_2_, and Z_3_ (respectively) are derived by repeatedly applying a dilation operator (⊕) on the area enclosed by B (denoted by α(B)) using a square structuring element D with a dimension d_max_ as follows:

$$Z_{1}=\alpha (B)⊕D+\alpha (B)-Z_{0};\;Z_{2}=Z_{1}⊕D-Z_{0};\;Z_{3}=Z_{2}⊕D-Z_{0}$$ (4)

We use these zones to compare OSM editing activity within the camp versus its 3 surrounding zones (Z_1_, Z_2_, Z_3_) during a ±4 month period around the strongest extremum point extracted from the weekly data, and examined at the daily granularity for each camp. The temporal window used for each refugee camp is shown in Table 3 with the results from this analysis summarized in S3 File. These results exhibit a sharp decline in editing activity along the transition from the camps outwards, to their surrounding zones. On average, for five of the eight camps the drop in number of OSM edits from Zone Z_0_ to Z_1_ was 93%. The remaining three camps, namely Kakuma, Calais and Bidibidi, have edits in Z_1_ exceeding edits within Z_0_. In the case of Calais, this finding can be explained by the camp’s neighboring synonymous city being part of the Calais Z_1_ zone. Similarly, in the case of Bidibidi, the increase in the number of OSM edits can be attributed to the fact that Bidibidi is surrounded by other refugee camps that are characterized by substantial edit activity, and are included in Bidibidi’s corresponding Zones Z_1_ to Z_3_.

**Table 3:** Temporal window (±4 month period) around the strongest extremum point extracted from the weekly data at the daily granularity.

Site	Start date	Stop date
Dadaab	10/16/14	6/15/15
Kakuma	10/6/16	6/5/17
Nyarugusu	1/25/15	9/24/15
Calais	5/7/15	1/6/16
Yida	9/25/15	5/24/16
Bidibidi	9/23/16	5/22/17
Oncupinar	1/30/16	9/29/16
Zaatari	5/19/16	1/18/17

The drop in the number of OSM edits becomes even more pronounced when considering the normalized edit metrics per area for each zone. On average, for the eight camps studied here, Z_1_, Z_2_, and Z_3_ zones cover an area that is 18, 54, and 108 times larger than the area of Z_0_, respectively. In order to account for these zone area variations, the number of OSM edits is normalized to be per km^2^, as shown in S3 File. With the exception of Kakuma, the data shows that on average the number of OSM edits per km^2^ drops as one moves from Z_0_ outwards by 96.37% (Z_1_), 97.96% (Z_2_), and 98.42% (Z_3_). This pattern was also similar for Kakuma, however, the drop in the number of edits per km^2^ was much lower, moving from Z_0_ outwards by 2.71% (Z_1_), 63.99% (Z_2_), and 81.91% (Z_3_). These results, therefore, support the notion that the camps are indeed serving as local activity hotspots, attracting OSM edits from the corresponding volunteer community beyond what would be expected by their surrounding areas.

Similar to the number of OSM edits, an analysis of the number of contributors per km^2^ was carried out, as summarized in S3 File. These results exhibit a trend similar to the one found in the number of OSM edits per km^2^: the number of contributors per km^2^ also drops as one moves from Z_0_ outwards by 95.07% (Z_1_), 97.59% (Z_2_), and 98.09% (Z_3_). Interestingly, in the case of Calais, the number of edits per km^2^ is increasing moving from Z_0_ outwards. Once again, this reversal in trend can be explained by the proximity of this refugee camp to a large urban area in a developed country.

In order to complete the saliency analysis, S3 File also lists the number of individual camp contributors who were active in the surrounding zones. As can be seen from these results, overall, the number of contributors who were active in the each of the camp zones (zone Z_0_) who were also active in other zones is approximately 40% (the values range between 25%-50%), further suggesting the role of camps as local OSM edit activity hotspots.

### Public awareness versus OSM and Wikipedia edit activity

In order to assess the possible relationship between news media coverage and digital activism in OSM, we compare OSM edit activity to three additional data sources, namely Wikipedia edits, Google News and Google Trends for each camp using the progressive refinement approach described earlier. As stated previously, in the context of this comparison, Google News is used as an indicator of media coverage, conveying how frequently a refugee camp appeared in news. In contrast, Google Trends is used as an indicator of broad public interest in this camp. Finally, Wikipedia edit activity represents an example of a non-geographic form of digital activism.

**Fig 5:**
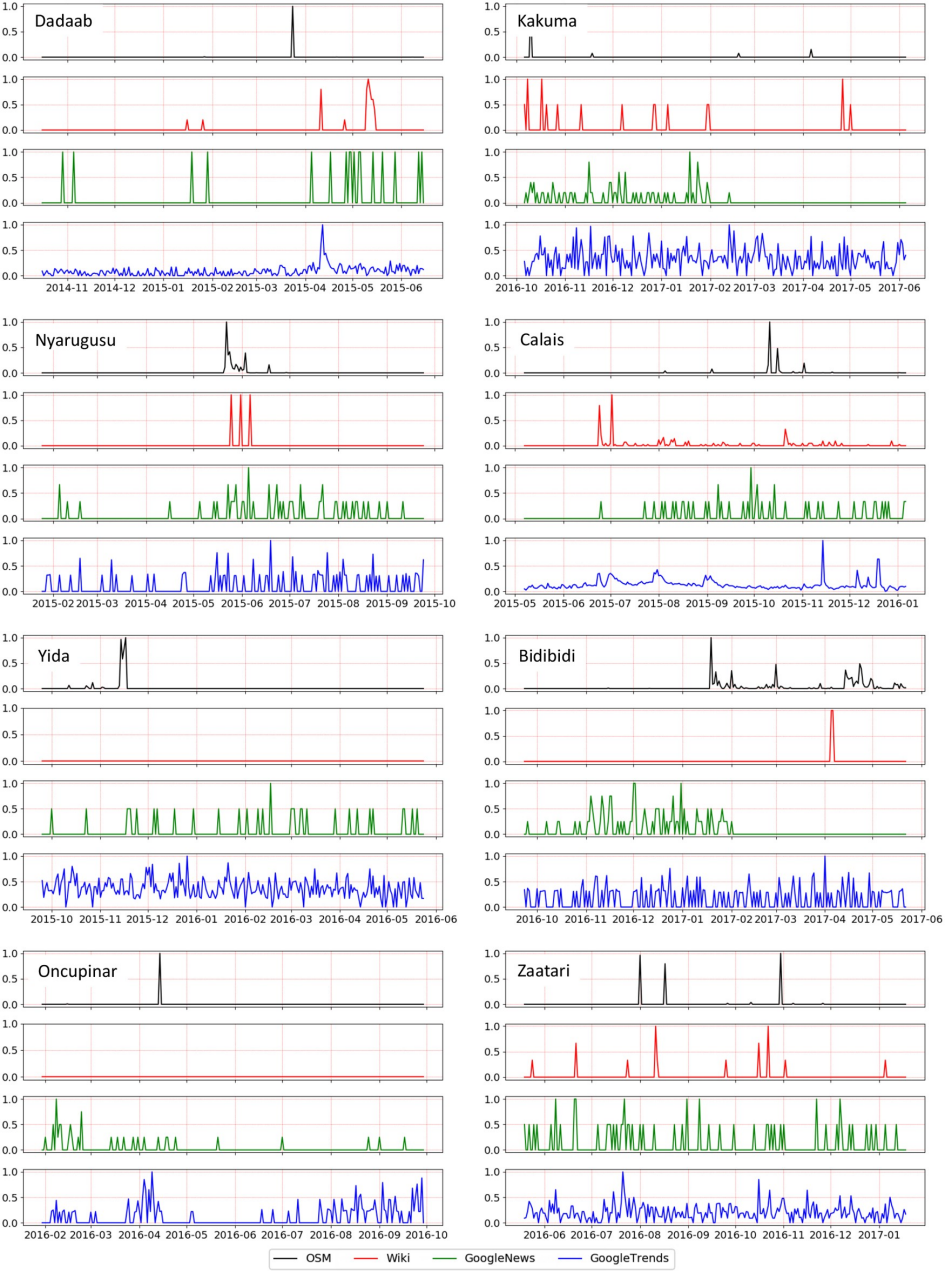
OSM, Wikipedia, Google News, and Google Trends time series during a ±4 months period around the strongest extremum point of each camp. The figures show that whereas OSM and Wikipedia entries tend to come in bursts, Google News and Trends display a more sustained type of activity.

**Fig 6:**
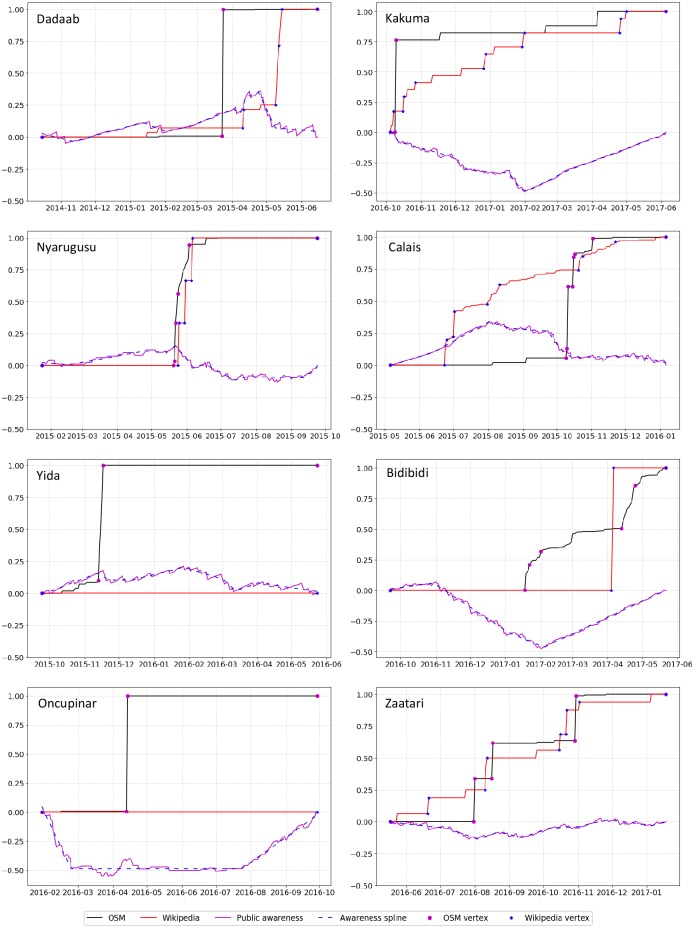
The public awareness curve versus the cumulative OSM and Wikipedia edit activity during a ±4 months period around the strongest extremum point of each camp. For camps such as Nyarugusu, OSM and Wikipedia bursts overlap with public awareness surplus. In other camps, such as Bidibidi, OSM edit activity bursts coincide with public awareness deficit.

Fig 5 shows the time series of all four data sources (OSM edits, Wikipedia edits, Google News items, and Google Trends indicator) for each of the eight camps during a ±4 months period around the strongest extremum point of each camp (at the weekly temporal granularity–Table 3). Based on these time series data, the public awareness curve was calculated (as the cumulative difference of Google Trends and Google News activity) for each camp and the strongest extremum points were detected. Then, using the progressive refinement approach presented earlier, we examined the relationship between the public awareness curve and the OSM and Wikipedia edit activity in a time window of ±4 months around the extremum point. Fig 6 depicts the public awareness curve (magenta line) along with the cumulative OSM and Wikipedia edit activity (black and red lines, respectively). In these graphs we also show splines (dashed blue lines) fitted to the public awareness curves to better visualize the overall trends in these curves. In the context of our approach, because we analyze extreme cases of public awareness, any significant deviation between the two input variables (i.e. Google Trends and Google News) at the daily analysis level is viewed as a possible predictor of activism activity.

As the graphs in Fig 6 show, digital activism bursts tend to be associated with periods of consistent build-up of surplus or deficit in public awareness. This tendency is particularly evident in the Dadaab, Nyarugusu and Yida camps, where OSM edit activity bursts tend to overlap with public awareness surplus, and in the Bidibidi camp, where OSM edit activity bursts coincide with public awareness deficit. Such patterns of activity are also evident with respect to Wikipedia edits for some camps: for example, Dadaab, Nyarugusu and Bidibidi. However, in other camps, namely Kakuma, Calais and Zaatari, Wikipedia edits exhibit a rather prolonged sustained effort of edit activity compared to the activity bursts in OSM (note that for Yida and Oncupinar no Wikipedia edits were made during the ±4 months period around the strongest extremum point).

To further examine the association between public awareness trends and OSM and Wikipedia edit activity, we derived the time gap between the most significant extremum point in OSM and Wikipedia extremum points to the closest extremum point in the public awareness curve of each camp at the three temporal granularity levels. The time gaps that were found at the monthly, weekly, and daily time granularities as a result of the progressive refinement process along with the range of time gap values across the eight camp sites are provided in Table 4. As can be seen from this table, at the finest (daily) temporal granularity, the average time gap is approximately between 11 and 12 days.

**Table 4:** Summary of the time gap values derived at the monthly, weekly, and daily levels for OSM and Wikipedia.

Platform	Time granularity units	Average time gap (and range)
OSM	Monthly	104.6 (0, 334)
	Weekly	43.0 (0, 126)
	Daily	10.8 (1, 32)
Wikipedia	Monthly	108.8 (0, 304)
	Weekly	44.3 (0, 133)
	Daily	12.0 (1, 27)

## Discussion

Today’s age of the participatory news consumer [104] has been steadily blurring the lines between digital content consumption and production [105,106]. An emerging manifestation of this change is the bridging of the gap between the omnipresence of news in one’s daily life and one’s resulting expression of activism [107,108]. Such expressions of activism have previously been studied in the context of the shaping of public policy (e.g. [109,110]), political campaigns (e.g. [62]), environment (e.g. [111]) and climate change (e.g. [112]) issues. However, little is still known about the impact that news media coverage has on digital activism, especially as it relates to online crowdsourcing platforms such as OSM and Wikipedia.

Our objective in this paper was to advance our understanding of the complex interrelationships that link media coverage and digital activism by focusing in particular on the geographical dimension of news media and the manifestation of digital activism—in particular edit activity—in both geographic and non-geographic crowdsourcing platforms. In order to pursue this goal, we used refugee camps as a test case. As noted earlier, refugee camp sites are particularly suitable for this objective due to their conceptual alignment to the motivational factors that drive volunteerism, and due to their distinct geographical locations, that render their OSM and Wikipedia contributions exclusively related to the camps themselves. Our analysis focused on two interrelated themes, namely the saliency of refugee camps as geographically distinct subjects of digital activism, and the possible co-occurrences between public awareness trends and digital activism. In both cases edit activity of contributors in OSM and Wikipedia was considered to be a manifestation of digital activism.

Considering the issue of saliency, we examined the OSM edit activity in each camp site and a set of three surrounding zones, both in terms of the number of edits and in terms of unique contributors. It was found that, in general, both the total number of edits and the number of edits per km^2^ drops substantially around camp sites compared to the edit activity within them. Additionally, a similar decay was found with respect to the number of unique contributors that were engaged in edit activities in camp sites versus the zones surrounding these sites. These results suggest that refugee camp sites tend to serve as geographically salient features that attract purposeful digital activism. Moreover, the decay in the number of unique contributors around the periphery of the camp sites suggest that the camp sites become salient objects of awareness to which OSM contributors pay specific attention. These findings give rise to the idea that the geographic saliency and awareness saliency are interdependent in the context of digital activism.

Focusing on the notion of awareness saliency and digital activism, we then explored the relationship between public awareness and evidence of digital activism related to refugee camps. Using a public awareness measure that was derived from Google News and Google Trends, we compared trend changes in public awareness to patterns of edit activity in the OSM and Wikipedia crowdsourcing platforms. Our findings indicate that in these platforms digital activism bursts tend to take place during periods of build-up of public awareness surplus or deficit, with an average time gap of approximately 11 to 12 days from extremum points in OSM and Wikipedia activity curves to the closest extremum point in the public awareness curve. It is important to note that the average time gap values were consistent across the two crowdsourcing platforms for all tree time granularities that were examined (namely monthly, weekly, and daily). However, our analysis shows that these two platforms do not always share similar activity patterns. Specifically, the results suggest that OSM edit activity within refugee camps tends to be concentrated in distinct bursts, while Wikipedia edit activity is often characterized by a gradual sustained edit activity effort. This difference suggests that while the user communities in both platforms are potentially exposed to the same public awareness trends, the response of each community may not be the same.

While our analysis did not address directly the issue of motivation, the results of our public awareness analysis highlight the multifaceted nature of motivation in the context of crowdsourcing. Specifically, our results indicated that edit activity bursts can occur during periods of sustained surplus or deficit in public awareness. These seemingly contradicting findings can be explained by two complementary theories in mass communication related to activism, namely agenda setting theory [99] and corrective action theory [113]. A manifestation of the former is the finding that periods of consistent public awareness surplus lead to increased saliency of the corresponding refugee camps as attention artifacts, which in turn lead to edit activity bursts (as is the case with Dadaab, Nyarugusu, and Yida). A manifestation of the latter is the finding that periods of consistent public awareness deficit (as is the case with the rest of the camps), which increases the saliency of the camp sites as attention artifacts due to the perceived lack of coverage of the topic in the news media, and leads to edit activity.

**Fig 7:**
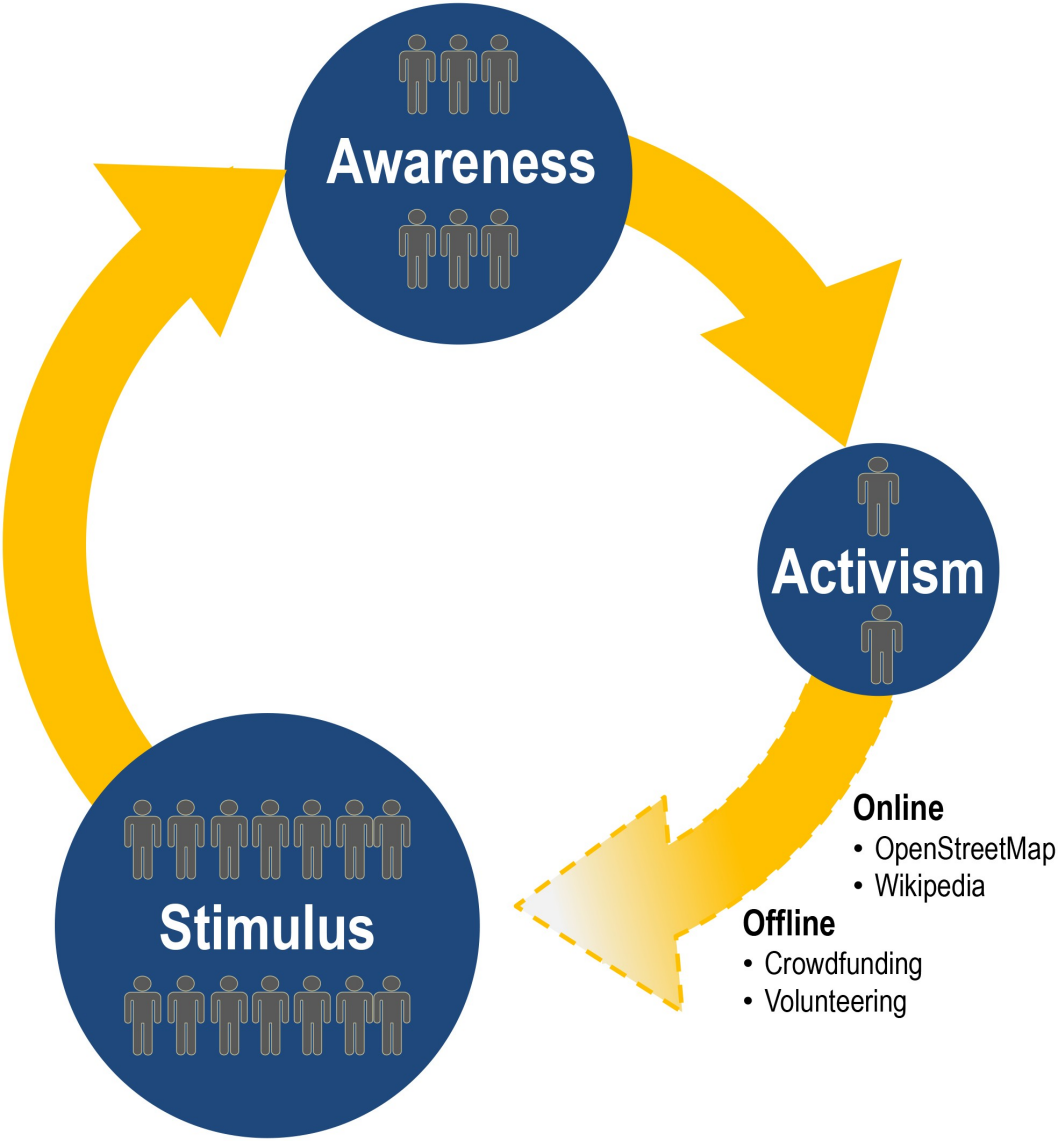
Stimulus-awareness-activism (SA^2^) framework.

Combined, these results suggest the potential of a novel stimulus-awareness-activism (SA^2^) framework in today’s participatory digital age. This framework, as presented in Fig 7, is built on three primary constructs: (1) stimulus, (2) awareness, and (3) activism. In this framework, *stimulus* is provided by news media coverage of a specific topic (expressed through Google News metrics). We argue that over time, such coverage leads to *awareness*, whereby the public seeks additional information on the topic (expressed through Google Trends metrics). When awareness grows faster than news coverage a build-up of awareness surplus occurs: a topic resonates with the public, and in a sense goes viral. When awareness growth lags in comparison to news coverage awareness deficits occurs: a topic fails to capture the public’s interest and slowly fades away. Build-ups of awareness surplus or deficits lead to activism. Such activism can be manifested either offline (e.g. participating in crowdfunding efforts or volunteering for a non-government organization) or online (e.g. participating in OSM or Wikipedia activities). One could reasonably expect that this process is cyclical in nature, as activism is likely to lead to increased news coverage, providing renewed stimulus and creating a feedback loop in this stimulus-awareness-activism (SA^2^) framework. In terms of the individuals involved in such activities, it’s important to note that while a large population of individuals may be exposed to the stimulus, only a portion of this population may develop awareness, and an even smaller portion will engage in activism. Additionally, it is important to point out that new individuals may become exposed and develop awareness as the topic gains saliency in the news awareness ecosystem.

In this paper, we studied the framework that links news coverage to awareness and activism by focusing in particular on the question of geographical saliency. Certain news stories tend to have a specific geographic dimension associated with them, and refugee camps are an excellent example of this, both explicitly and implicitly. *Explicitly* because these camps are physical constructs and have a specific location that they occupy (and in some cases are even named after that location), while *implicitly* because they are stops along geographical pathways that take the refugees from an origin location (e.g. the homeland that they had to abandon) to a destination location (their intended final destination). Accordingly, geographical saliency is rather prominent when considering these types of stories. However, geography is also prominent in most other news stories [114], just like for example geographical content is prominent in the vast percentage of Wikipedia entries [115].

As with any study, this study has some noteworthy limitations. Here, we highlight several such limitations that could be further investigated and refined. The first relates to the number of refugee camps studied. In our paper, although we selected refugee camps from around the world, only 8 camps were used. However, a larger cross-section of camps would be useful in exploring the relationship in news media coverage and digital geo-activism in greater breadth. Such research can also benefit from a much longer-term study of these variables, which can further be used to better understand the movement of these variables overtime and their possible association with other exogenous factors (e.g. crisis events). Second, while our analysis was done in the context of refugee camps, further analysis is required in order to explore whether similar patterns can be observed in other contexts (e.g. natural and manmade disasters, and disease outbreaks). Third, as noted above, the examination of motivational factors is not a straightforward process and requires further investigation (e.g. large-scale surveys of the motivational factors of OSM contributors).

Another, fourth, issue relates to the focus of this study only on the news awareness ecosystem in the English language, primarily due to the overwhelming pervasiveness of English in online content compared to other languages [116]. As the behavior of the news awareness ecosystem in other languages may be different, further analysis of the possible relationship between public awareness and digital activism across different languages is needed. A fifth issue we highlight concerns the use of the volume of Google News items in the public awareness measure used in this work. By considering only the volume and not the content or the impact of each news item our approach takes a simplistic view in which all news items are regarded as equal. However, in practice it is possible that some news items (or news outlets) may become more influential than others, which may result in a different pattern of digital activism. Examining this issue requires a separate line of inquiry that involves the development of appropriate measures for estimating the influence of news items as well as content analysis.

A sixth related issue is that our study only considers extreme bursts of activity in public awareness that tend to trigger digital geo-activism. However, the input variables used in determining such public awareness are expected to be in a perpetual state of fluctuation, with their own unique circadian patterns and influenced by various factors such as seasonality and crisis situations, among others. A more in-depth study analyzing these specific patterns would therefore be of interest. Another related limitation of our study is that for some camps, namely Yida and Oncupinar, there were no Wikipedia edits for these camps for the specific search window used when examined at the daily granular level. In the case of Oncupinar, recent reports have shown that Wikipedia editing access in Turkey was blocked by government authorities in April 2017 [117]. This period, however, is beyond our study period. Nevertheless, it is difficult to assess the impact that such actions may have on inactive periods of edit activity in online platforms such as Wikipedia since for example, technology measures exist that may nullify their effect (e.g. [118–119]). Recent studies, for example, have shown that digital censorship may also have the opposite effect, that is, there is an increase drive to access more information, and thus innovative ways to overcome such restrictions emerge (e.g. [120]).

Further work could also address the degree to which geographical saliency drives digital activism in news stories that relate to geographical areas that are not as monothematic as refugee camps, but rather are often featured in news stories for a wide variety of issues. For example, a megacity may find itself in the news following a major disaster, yet at the same time, it may also be featured for the numerous other activities/issues that are associated with it. Studying such multi-thematic geographical areas will allows us to further refine the SA^2^ framework. Such refinements will allow us to devise more effective communication campaigns that will harness the power of the crowd in an organized manner to build responses to societal needs, such as mapping uncharted parts of the Sub-Saharan Africa to better study the birth and spread of exotic diseases at the human-environment interface. Even more importantly, such studies will offer us a better understanding of how our societies function across the cyber-physical news awareness ecosystem that is becoming the prevailing paradigm when interacting with the news.

## Supporting information

S1 File Population references for refugee camps. (DOCX)

S2 File OSM edits, Wikipedia edits and Google News articles at the daily level for the period 01/01/2010 to 05/31/2017. (DOCX)

S3 File OSM edits within camps and surrounding areas (±4 month period) around the strongest extremum point extracted from the weekly data at the daily granularity. (DOCX)
