# Supplementary material for: News coverage, digital activism, and geographical saliency: A case study of refugee camps and volunteered geographical information
Source: PLoS One. 2018 Nov 8;13(11):e0206825. doi: 10.1371/journal.pone.0206825 (PMC6226103; doi:10.1371/journal.pone.0206825)
Supplement: S1 File — (DOCX) [file pone.0206825.s001.docx]

S1 Table. Population references for refugee camps

| Camp | Population references |
| --- | --- |
| Dadaab | http://www.unhcr.org/ke/dadaab-refugee-complex |
| Kakuma | http://reliefweb.int/sites/reliefweb.int/files/resources/56243.pdf |
| Nyarugusu | https://data2.unhcr.org/en/documents/details/58872 |
| Calais | https://reliefweb.int/report/france/voices-calais-introduction  http://www.lgsmun.org/docs/unhcr.pdf  http://www.aljazeera.com/news/2016/09/france-close-calais-camp-relocate-refugees-weeks-160924194133102.html  http://www.express.co.uk/news/uk/703213/calais-knife-edge-9-000-british-bound-migrants-jungle-shanty-town-cross-channel-France  http://www.france24.com/en/20160819-france-calais-spectacular-increase-migrants-refugees-jungle-camp |
| Yida | http://data.unhcr.org/SouthSudan/region.php?id=26  http://www.reachresourcecentre.info/system/files/resource-documents/SSD%20Report%2C%20Yida%20Refugee%20Camp%20Social%20Profile%2C%20Unity%20State%2C%20November%202012.pdf |
| Bidibidi | https://www.unicef.org/appeals/files/UNICEF_Uganda_Humanitarian_Situation_Report_March_2017.pdf  https://data2.unhcr.org/ar/documents/download/42015  http://www.worldvision.org.uk/news-and-views/latest-news/2017-news/may/world-vision-alarmed-staggering-number-unaccompanied-south-s/ |
| Oncupinar | https://www.pri.org/stories/2016-02-17/one-turkish-town-has-done-so-much-syrian-refugees-it-s-nobel-peace-prize  http://www.stalbertgazette.com/article/we-shouldnt-take-any-refugees-20151209 |
| Zaatari | http://data.unhcr.org/syrianrefugees/settlement.php?id=176 |
