# Supplementary material for: News coverage, digital activism, and geographical saliency: A case study of refugee camps and volunteered geographical information
Source: PLoS One. 2018 Nov 8;13(11):e0206825. doi: 10.1371/journal.pone.0206825 (PMC6226103; doi:10.1371/journal.pone.0206825)
Supplement: S2 File — (DOCX) [file pone.0206825.s002.docx]

S2 Table. OSM edits, Wikipedia edits and Google News articles at the daily level for the period 01/01/2010 to 05/31/2017.

| **Camps** | **Total OSM Edits** | **Wikipedia** | **Google News** |
| --- | --- | --- | --- |
| Dadaab | 31,283 | 334 | 227 |
| Kakuma | 11,147 | 139 | 489 |
| Nyarugusu | 74,416 | 16 | 166 |
| Calais | 5,965 | 483 | 162 |
| Yida | 84,053 | 4 | 253 |
| Bidibidi | 198,916 | 11 | 120 |
| Oncupinar | 708 | 16 | 103 |
| Zaatari | 69,981 | 164 | 290 |
