# Supplementary material for: News coverage, digital activism, and geographical saliency: A case study of refugee camps and volunteered geographical information
Source: PLoS One. 2018 Nov 8;13(11):e0206825. doi: 10.1371/journal.pone.0206825 (PMC6226103; doi:10.1371/journal.pone.0206825)
Supplement: S3 File — (DOCX) [file pone.0206825.s003.docx]

S3 Table. OSM edits within camps and surrounding areas (±4 month period) around the strongest extremum point extracted from the weekly data at the daily granularity.

|  |  | **Camp zone** | **Surrounding Zones (Distance in km)** | | |
| --- | --- | --- | --- | --- | --- |
| **Dadaab** | **Distance (km)** | **Z0** | **Z1 (5)** | **Z2 (10)** | **Z3 (15)** |
|  | **Contributors (camp active)** | 4 | 2(1) | 2(1) | 3(1) |
|  | **Total edits** | 6,355 | 232 | 232 | 371 |
|  | **Contributors/km^2^** | 9.82 x10^-2^ | 0.33 x10^-2^ | 0.16 x10^-2^ | 0.14 x10^-2^ |
|  | **Edits per km^2^** | 1.56 x10^2^ | 38.70 x10^-2^ | 18.40 x10^-2^ | 17.48 x10^-2^ |
|  |  |  | | | |
| **Kakuma** | **Distance (km)** | **Z0** | **Z1 (3)** | **Z2 (6)** | **Z3 (9)** |
|  | **Contributors (Camp active)** | 4 | 2 (1) | 3 (2) | 3 (2) |
|  | **Total edits** | 17 | 191 | 208 | 208 |
|  | **Contributors/km^2^** | 62.50 x 10^-2^ | 2.71 x 10^-2^ | 1.38 x 10^-2^ | 0.69 x 10^-2^ |
|  | **Edits per km^2^** | 2.66 | 2.58 | 95.66 x 10^-2^ | 48.04 x 10^-2^ |
|  |  |  | | | |
| **Nyarugusu** | **Distance (km)** | **Z0** | **Z1 (8)** | **Z2 (16)** | **Z3 (24)** |
|  | **Contributors (camp active)** | 40 | 21 (12) | 34 (13) | 57 (13) |
|  | **Total edits** | 72240 | 11356 | 25102 | 40239 |
|  | **Contributors/km^2^** | 1.54 | 4.43 x 10^-2^ | 2.35 x 10^-2^ | 1.94 x 10^-2^ |
|  | **Edits per km^2^** | 27.89 x10^2^ | 23.95 | 17.35 | 13.72 |
|  |  |  | | | |
| **Calais** | **Distance (km)** | **Z0** | **Z1 (1)** | **Z2 (2)** | **Z3 (3)** |
|  | **Contributors (camp active)** | 9 | 10 (4) | 16 (4) | 21 (4) |
|  | **Total edits** | 1117 | 1709 | 3462 | 5461 |
|  | **Contributors/km^2^** | 14.75 | 1.27 | 66.09 x 10^-2^ | 42.77 x 10^-2^ |
|  | **Edits per km^2^** | 18.31 x 10^2^ | 216.60 | 143 | 111.22 |
|  |  |  |  |  |  |
| **Yida** | **Distance (km)** | **Z0** | **Z1 (6)** | **Z2 (12)** | **Z3 (18)** |
|  | **Contributors (camp active)** | 5 | 7 (2) | 9 (2) | 9 (2) |
|  | **Total edits** | 68064 | 2251 | 4448 | 17509 |
|  | **Contributors/km^2^** | 24.51 x10^-2^ | 2.52 x10^-2^ | 1.08 x10^-2^ | 0.54 x10^-2^ |
|  | **Edits per km^2^** | 33.36 x10^2^ | 8.11 | 5.32 | 10.41 |
|  |  |  | | | |
| **Bidibidi** | **Distance (km)** | **Z0** | **Z1 (23)** | **Z2 (46)** | **Z3 (69)** |
|  | **Contributors (camp active)** | 217 | 235 (80) | 251 (81) | 258 (81) |
|  | **Total edits** | 191904 | 259317 | 270264 | 272911 |
|  | **Contributors/km^2^** | 60.12 x10^-2^ | 4.10 x10^-2^ | 1.67 x10^-2^ | 0.90 x10^-2^ |
|  | **Edits per km^2^** | 531.74 | 45.20 | 17.92 | 9.52 |
|  |  |  | | | |
|  | **Distance (km)** | **Z0** | **Z1 (2)** | **Z2 (4)** | **Z3 (6)** |
| **Oncupinar** | **Contributors (camp active)** | 2 | 1 (1) | 4 (1) | 8 (1) |
|  | **Total edits** | 387 | 16 | 62 | 256 |
|  | **Contributors/km^2^** | 2.82 | 4.01 x 10^-2^ | 4.90 x10^-2^ | 11.37 x10^-2^ |
|  | **Edits per km^2^** | 545.07 | 64.10 x 10^-2^ | 75.98 x 10^-2^ | 3.64 |
|  |  |  | | | |
| **Zaatari** | **Distance (km)** | **Z0** | **Z1(3)** | **Z2 (6)** | **Z3 (9)** |
|  | **Contributors (camp active)** | 5 | 1 (1) | 1 (1) | 3 (2) |
|  | **Total edits** | 154 | 11 | 11 | 14 |
|  | **Contributors/km^2^** | 81.97 x 10^-2^ | 1.44 x 10^-2^ | 0.48 x10^-2^ | 0.71 x10^-2^ |
|  | **Edits per km^2^** | 25.25 | 15.91 x 10^-2^ | 5.24 x 10^-2^ | 3.31 x 10^-2^ |
